# Unlocking the Full Potential of SGLT2 Inhibitors: Expanding Applications beyond Glycemic Control

**DOI:** 10.3390/ijms24076039

**Published:** 2023-03-23

**Authors:** Mahmoud E. Youssef, Galal Yahya, Mihaela Simona Popoviciu, Simona Cavalu, Marwa A. Abd-Eldayem, Sameh Saber

**Affiliations:** 1 Department of Pharmacology, Faculty of Pharmacy, Delta University for Science and Technology, Gamasa 11152, Egypt; 2 Department of Microbiology and Immunology, Faculty of Pharmacy, Zagazig University, Al Sharqia 44519, Egypt; 3 Faculty of Medicine and Pharmacy, University of Oradea, P-ta 1 Decembrie 10, 410087 Oradea, Romania; 4 Department of Pharmacology and Biochemistry, Faculty of Pharmacy, Horus University, New Damietta 34518, Egypt

**Keywords:** SGLT2 inhibitors, cardiovascular effects, cardiorenal effects, cancer, bone minerals, cognitive effects, metabolism

## Abstract

The number of diabetic patients has risen dramatically in recent decades, owing mostly to the rising incidence of type 2 diabetes mellitus (T2DM). Several oral antidiabetic medications are used for the treatment of T2DM including, α-glucosidases inhibitors, biguanides, sulfonylureas, meglitinides, GLP-1 receptor agonists, PPAR-γ agonists, DDP4 inhibitors, and SGLT2 inhibitors. In this review we focus on the possible effects of SGLT2 inhibitors on different body systems. Beyond the diabetic state, SGLT2 inhibitors have revealed a demonstrable ability to ameliorate cardiac remodeling, enhance myocardial function, and lower heart failure mortality. Additionally, SGLT2 inhibitors can modify adipocytes and their production of cytokines, such as adipokines and adiponectin, which enhances insulin sensitivity and delays diabetes onset. On the other hand, SGLT2 inhibitors have been linked to decreased total hip bone mineral deposition and increased hip bone resorption in T2DM patients. More data are needed to evaluate the role of SGLT2 inhibitors on cancer. Finally, the effects of SGLT2 inhibitors on neuroprotection appear to be both direct and indirect, according to scientific investigations utilizing various experimental models. SGLT2 inhibitors improve vascular tone, elasticity, and contractility by reducing oxidative stress, inflammation, insulin signaling pathways, and endothelial cell proliferation. They also improve brain function, synaptic plasticity, acetylcholinesterase activity, and reduce amyloid plaque formation, as well as regulation of the mTOR pathway in the brain, which reduces brain damage and cognitive decline.

## 1. Introduction

Diabetic patients have increased considerably in recent decades, mostly due to the increase in the frequency of type 2 diabetes mellitus (T2DM). In addition to health concerns, this is related to severe economic and sociologic challenges [1]. Treatment of diabetes is costly, and the amount spent on it each year is increasing. On the market, there are several antidiabetic drugs that may be used alone or in combination. The mechanism of action of each drug is distinct, and it can alter depending on a range of conditions, including dose.

Antidiabetic drugs are intended to control glucose metabolism, with the primary objective of lowering blood glucose levels in a non-specialized sense. As a result, most of them may be able to cure other diseases, particularly obesity, which is a major factor in developing diabetes mellitus (DM) [2]. As a result, the complexity of available compounds, their methods of action, and biological activities have become hot topics of debate in a variety of human health sectors, including cardiovascular, renal, neurologic, and even cancer diseases [3]. Because diabetes is such a complex disease, it necessitates a thorough examination when seeking new targets to treat it or the mechanism of action of medications with possible antidiabetic properties [4]. Furthermore, some, if not all, of these chemicals have the capacity to modify cellular metabolism in ways that may be beneficial in certain organs but harmful in others. This is a perplexing issue that obstructs the process [5].

## 2. Sodium–Glucose Transporters (SGLTs)

Sodium–glucose cotransporters (SGLTs) are a family of transmembrane proteins that play a crucial role in the transport of glucose and sodium ions across cell membranes. SGLTs are expressed in various tissues, including the small intestine, renal tubules, and the heart. There are three families of glucose transporters (encoded by *SLC2A, SLC5A, SLC50A*). In humans, SGLTs are encoded by *SLC5A*. There are 12 genes of *SLC5A*, and they have been detected in different tissues each with a specific distribution and function.

### 2.1. SLC5A1 (SGLT1)

SGLT1 is primarily expressed in the small intestine, where it plays a crucial role in the absorption of glucose from the gut lumen [6]. It is also present in the renal proximal tubules, where it contributes to glucose reabsorption from the filtrate. SGLT1 is a high-affinity, low-capacity transporter that transports glucose and galactose in a 1:1 ratio with sodium ions. It is a secondary active transporter that uses the electrochemical gradient of sodium ions to drive the transport of glucose against its concentration gradient. The activity of SGLT1 is regulated by various factors, including insulin, glucagon-like peptide-1 (GLP-1), and sodium ions [7].

### 2.2. SLC5A2 (SGLT2)

SGLT2 is mainly expressed in the renal proximal tubules, where it is responsible for the reabsorption of about 90% of filtered glucose. It is a high-capacity, low-affinity transporter that transports glucose and other monosaccharides in a 1:1 ratio with sodium ions. SGLT2 is also a secondary active transporter that uses the electrochemical gradient of sodium ions to drive the transport of glucose against its concentration gradient [8]. The activity of SGLT2 is regulated by various factors, including insulin, glucagon, and sodium ions [9].

### 2.3. SLC5A4 (SGLT3)

SGLT3 (SAAT1) is expressed in the heart and brain, where it is believed to play a role in glucose sensing and regulation of insulin secretion. Unlike SGLT1 and SGLT2, SGLT3 is a low-affinity, high-capacity transporter that transports glucose and other monosaccharides in a 1:1 ratio with sodium ions [10]. However, the physiological role of SGLT3 in glucose metabolism is still not fully understood, and its potential as a drug target for the treatment of diabetes is currently being investigated [11]. The activity of SGLT3 is regulated by various factors, including ATP, sodium ions, and intracellular pH [12].

### 2.4. SLC5A9 (SGLT4)

SGLT4, a low-affinity sodium–glucose cotransporter, belongs to the solute carrier family *SLC5* [13]. It is hypothesized to facilitate the transport of mannose, fructose, and 1,5-anhydroglucitol (1,5-AG) within the kidney. Immunolocalization studies have revealed the presence of SGLT4 in the luminal membrane of intestinal epithelial cells. Expression levels of SGLT4 have been found to be comparatively high in the small intestine and kidney, with moderate expression observed in the liver [14].

### 2.5. SLC5A10 (SGLT5)

SGLT5 is a sodium–glucose cotransporter that is mainly expressed in the kidney, where it is responsible for the reabsorption of 1,5-AG, a dietary polyol that is a marker of glycemic control. SGLT5 is also expressed in other tissues, such as the liver, intestine, and testis, where it may have other functions [13]. SGLT5 mutations have been associated with congenital neutropenia, a condition characterized by low levels of white blood cells. SGLT5 inhibitors are a potential new class of drugs that could lower blood glucose levels by increasing urinary excretion of 1,5-AG and glucose [15].

### 2.6. SLC5A11 (SGLT6)

SGLT6, also known as sodium myo-inositol transporter 2 (SMIT2), is a sodium–glucose cotransporter that is mainly expressed in the small intestine and brain, where it is responsible for the reabsorption of myo-inositol and glucose. Myo-inositol is a sugar alcohol that is involved in various cellular processes, such as signal transduction, osmoregulation, and lipid synthesis [16]. SGLT6 mutations have been linked to congenital neutropenia and renal glucosuria. SGLT6 inhibitors are a potential new class of drugs that could lower blood glucose levels and body weight by increasing urinary excretion of myo-inositol and glucose [17].

### 2.7. SLC5A3 (SMIT)

SMIT (*SLC5A3*) is a protein-coding gene that encodes the sodium/myo-inositol cotransporter. The myo-inositol transporter SMIT (*SLC5A3*) accomplishes cellular accumulation of organic osmolytes and thus contributes to cell volume regulation [18]. SMIT1 is expressed in the heart [19].

### 2.8. SLC5A7 (CHT)

CHT (*SLC5A7*) is a protein-coding gene that encodes the high-affinity choline transporter. Transport of choline via the neuronal high-affinity choline transporter (CHT; *SLC5A7*) is essential for cholinergic terminals to synthesize and release acetylcholine (ACh) [20].

### 2.9. SLC5A6 (SMVT)

SMVT (*SLC5A6*) is a protein-coding gene that encodes the sodium-dependent multivitamin transporter. SMVT is a transporter for pantothenic acid (vitamin B5) and biotin (vitamin B7) at the blood–brain barrier. It is also a transporter for alpha lipoic acid and iodide [21]. Sodium-dependent multivitamin transporter (SMVT; product of the *SLC5A6* gene) is an important transmembrane protein responsible for the translocation of vitamins and other essential cofactors such as biotin, pantothenic acid, and lipoic acid [22].

### 2.10. SLC5A8 (SMCT1)

SMCT1 (*SLC5A8*) is a protein-coding gene that encodes the sodium-coupled monocarboxylate transporter 1 [23]. SMCT1 is a sodium-coupled (Na (+) -coupled) transporter for l-lactate and short-chain fatty acids. SMCT1 was originally identified as a putative tumor suppressor gene in the human colon [24]. SMCT1 mRNA and protein are expressed in the healthy human, rat, and mouse colon and the protein is localized at the colonocyte apical membrane [25].

### 2.11. SLC5A12 (SMCT2)

SMCT2 (*SLC5A12*) is a protein-coding gene that encodes the sodium-coupled monocarboxylate transporter 2. SMCT2 is a transporter for lactate and pyruvate. SMCT2 is structurally and functionally similar to SMCT1 (*SLC5A8*) [26].

### 2.12. SLC5A5 (NIS)

NIS (*SLC5A5*) is a protein-coding gene that encodes the sodium–iodide symporter or NIS. NIS protein transports iodide, a negatively charged version of iodine, into the cells of certain tissues. The NIS protein is found primarily in the thyroid gland [27]. The NIS gene encodes a highly specialized and efficient 80–90 kDa transmembrane glycoprotein that mediates the active transport of iodide from the bloodstream into the follicular cells [28].

## 3. Antidiabetic Medications

### 3.1. α-Glucosidases Inhibitors

Carbohydrates constitute an important part of most Western diets. Galactosidases, amylase, and α-glucosidases are enzymes that break down complex carbohydrates into monosaccharides [29]. Intestinal α-glucosidase inhibitors affect the rate of digestion of complex carbohydrates and disaccharides by competitively and irreversibly inhibiting glucosidases in the brush border membrane of enterocytes that line the intestinal villi [30]. As a result, carbohydrate digestion and monosaccharide absorption in the proximal jejunum are reduced or incomplete in the distal jejunum and ileum. As a result, the postprandial rise in plasma glucose levels is reduced and/or postponed. α-Glucosidase inhibitors provide the pancreatic β-cell additional time to respond to a rise in plasma glucose level by increasing insulin production [31].

### 3.2. Biguanides

In the 1920s, several glucose-lowering guanidine derivatives were developed because of the discovery that Galego Officinalis, a conventional plant that had previously been employed as a therapeutic for DM, contains a high concentration of guanidine [32]. These medications were basically forgotten once insulin became more widely available and used. Before the 1950s, biguanides were not researched for the treatment of DM. Phenformin, buformin, and metformin are three biguanides that have antidiabetic properties. They were discovered in the late 1950s. Metformin is now the most widely used biguanide globally since phenformin and buformin use have been phased out due to a high prevalence of lactic acidosis. For more than 50 years, type 2 diabetes has been treated with metformin [32].

In pancreatic β-cells, metformin has little influence on insulin release. Metformin has been shown in recent studies to block complex I of the electron transport chain, resulting in the stimulation of AMP-activated protein kinase (AMPK)-sensitive signaling [33]. Through phosphorylation of various critical proteins, AMPK controls cellular energy as well as glucose and lipid metabolism. Physiological consequences of raising its activity include stimulation of hepatic glucose production and fatty acid oxidation in the liver and muscles, reduction of hepatic glucose absorption, cholesterol and triglyceride synthesis, and lipogenesis. The plasma levels of the incretin hormone glucagon-like peptide 1 (GLP-1), which has antihyperglycemic properties through the activation of GLP-1 receptor, can increase when receiving metformin, which can lead to insulin resistance [34].

### 3.3. Sulfonylureas

Sulfonylureas were conceptualized and developed as insulin secretion boosters in the 1940s because of an inadvertent observation of hypoglycemic episodes when taking sulfonamides. In 1955, sulfonylureas became the first pharmacological alternative for treating non-insulin-dependent DM, alongside insulin injections. [35]. Tolbutamide, chlorpropamide, acetohexamide, and tolazamide were the first sulfonylureas to be developed. The newer second-generation drugs gliclazide, glipizide, and glibenclamide (glyburide) have essentially replaced these first-generation medications. Some researchers identify the most recent sulfonylurea, glimepiride, as a second-generation sulfonylurea, while others classify it as a third-generation sulfonylurea. These drugs affect sulfonylurea receptor 1 (SUR 1), which causes adenosine triphosphate (ATP)-dependent potassium channels to close [36]. Potassium channels play an important role in regulating insulin secretion from the beta cells of the pancreas. Pancreatic beta cells express ATP-sensitive potassium (KATP) channels that are needed for normal insulin secretion and are targets for drugs that modulate insulin secretion [37]. When glucose levels are high, glucose enters the beta cells and triggers a series of events that lead to the opening of potassium channels. This allows potassium ions to flow out of the cells, which depolarizes the cell membrane and triggers the release of insulin. Sulfonylureas block these potassium channels, which prevents potassium from leaving the cells and depolarizes the cell membrane, leading to an influx of calcium ions and triggering insulin release [38].

### 3.4. Meglitinides

Meglitinides are insulin secretagogues that operate on ATP-dependent potassium channels in the same way that sulfonylureas do. As a result, they have no impact on individuals who have been treated with sulfonylureas at their maximum therapeutic dose [39]. Given this, they provide an option for sulfonylurea therapy, although with almost identical drawbacks and a more complicated dosage regimen. Nateglinide, repaglinide, and mitiglinide are the three meglitinides being utilized in clinical practice. The first meglitinide analog, repaglinide, was a carbamoyl methyl benzoic acid derivative. It stimulates insulin production by shutting ATP-dependent potassium channels in pancreatic-cell membranes, although it is ineffective when extracellular calcium is not present [40].

### 3.5. GLP-1 Receptor Agonists

Because of its antihyperglycemic properties, the incretin hormone GLP-1 was first identified in the early 1990s as a potential target in the treatment of type 2 diabetes [41]. GLP-1 is produced in the small intestine by endocrine L-cells and released after meals, particularly those high in lipids and carbs. GLP-1 has direct effects on multiple organs when it is produced, thanks to interactions with receptors found in the pancreas, lung, heart, brain, stomach, kidneys, and intestines [42]. This connection causes adenylate cyclase to be activated and cAMP to be produced in pancreatic cells, which then stimulates insulin release via protein kinase. GLP-1, on the other hand, promotes insulin secretion through beta cells [43].

### 3.6. Peroxisome Proliferator-Activated Receptor γ (PPAR-γ) Agonists

The effect of thiazolidinediones, i.e., pioglitazone is mediated by a nuclear receptor called peroxisome proliferator-activated receptor, which controls the expression of a number of genes involved in adipocyte cell development and lipid and glucose metabolic pathways [44]. This class of medicines has the potential to increase insulin sensitivity and reduce insulin resistance. However, due to an increase in the likelihood of heart failure in high-risk patients, the popularity of prescribing this class of antidiabetic medicines has fallen. PPAR-γ ligands may influence the activity of the renin–angiotensin system by transcriptional regulation at both the mediator and receptor levels, in addition to lowering blood glucose levels [45].

### 3.7. Dipeptidyl Peptidase 4 (DDP4) Inhibitors

The DDP4 inhibitors are incretins that reduce blood glucose by boosting pancreatic insulin release [46]. The classical mechanism for DPP-4 inhibitors is that they inhibit DPP-4 activity in peripheral plasma, which prevents the inactivation of the incretin hormone GLP-1 in the peripheral circulation [47]. When compared to prior antidiabetic medicines, DDP4 inhibitors do not cause weight gain and are increasingly employed as a supplement to metformin-based first-line therapy [48]. However, in individuals with a high cardiovascular risk, this category may trigger heart failure or worsen the clinical course of patients with pre-existing left ventricular dysfunction.

### 3.8. Sodium–Glucose Transporter 2 (SGLT2) Inhibitors

**Figure 1:**
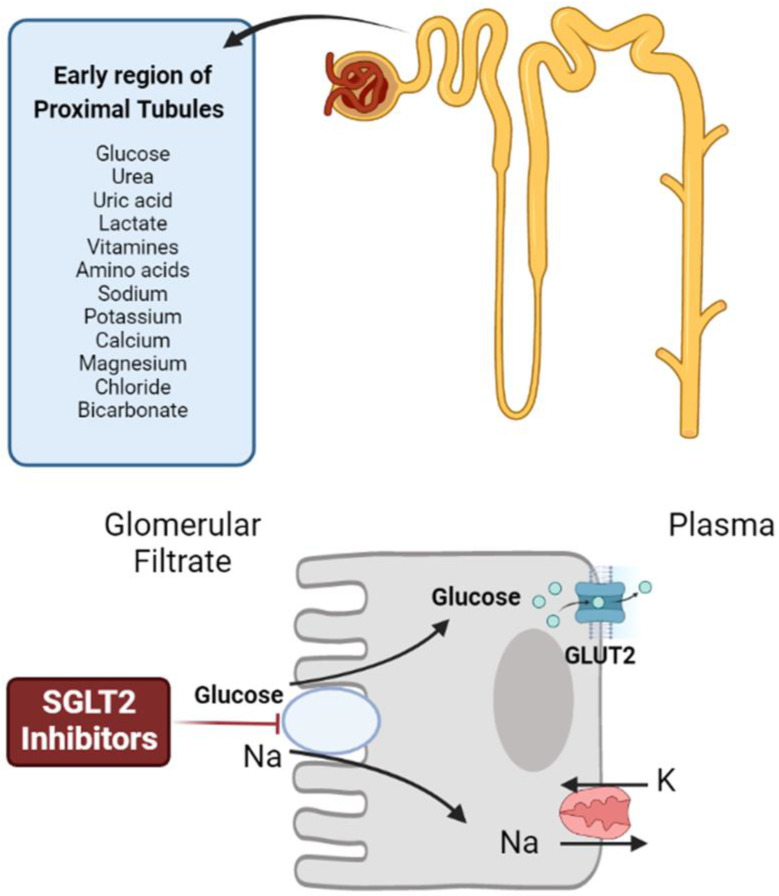
Mechanism of action of SGLT2 inhibitors.

The sodium–potassium adenosine triphosphate pump actively maintains a sodium gradient, and sodium–glucose cotransporters (SGLTs) play a role in blood glucose entry into cells across this gradient. SGLTs control the reabsorption of filtrated glucose from the glomeruli directly into the bloodstream at the renal level [49]. The SGLT2 is found in the early convoluted region of the proximal tubule and can process 90% of the filtrated glucose, whereas the SGLT1 subtype is expressed more distally and aids the kidney in reabsorbing the remaining 10% (Figure 1). Surprisingly, T2DM patients have much higher SGLT2 expression in their kidneys than do healthy individuals. Elevated plasma glucose levels in T2DM and increased reabsorption rate of glucose would stimulate the expression of SGLT2. Reduced glucose and salt absorption are linked to SGLT2 suppression [50].

SGLT2 inhibitors act by blocking the action of the SGLT2 protein in the kidneys. This protein is responsible for reabsorbing glucose back into the bloodstream, but SGLT2 inhibitors prevent this from happening, resulting in increased glucose excretion in the urine. As a result, blood glucose levels are lowered, which can improve glycemic control in people with type 2 diabetes. SGLT2 inhibitors also have other effects on the body, such as promoting weight loss and reducing blood pressure.

The initial theory behind the creation of SGLT2 inhibitors was to take advantage of the body’s renal glucose management by decreasing SGLT2-mediated glucose reabsorption from the proximal renal tubule, causing glycosuria and controlling hyperglycemia [51]. Canagliflozin was the first of four SGLT2 inhibitors (canagliflozin, dapagliflozin, empagliflozin, and ertugliflozin) authorized by the food and drug administration (FDA) to reduce blood glucose levels in persons with T2DM when combined with diet and exercise [52]. The initial theory behind the creation of these medications was to take advantage of the body’s renal glucose management by decreasing SGLT2-mediated glucose reabsorption from the proximal renal tubule, causing glycosuria and controlling hyperglycemia [51]. These insulin-independent antihyperglycemic medicines, on the other hand, are increasingly recognized as pleiotropic drugs that have considerable metabolic, cardiovascular, and renal advantages. Table 1 summarizes the general mechanisms of actions of different antidiabetic drugs.

## 4. Cardiovascular Effects of SGLT2 Inhibitors

SGLT2 inhibitors have been linked to cardiovascular benefits, including reducing collagen production, suppressing fibroblast activation, and reducing sympathetic overdrive [53,54]. SGLT2 inhibitors also increase natriuresis and osmotic diuresis, which lead to decreased renin production and reduced filtration rate, intraglomerular hydrostatic pressure, and blood pressure [55]. Other proposed mechanisms of SGLT2 inhibitors include nephron remodeling, relaxation of vascular smooth muscle cells, and weight loss independent of fluid contraction caused by glycosuria [56,57].

In the cardiovascular system, ATP plays a role in regulating vascular tone and contractility and can activate various signaling pathways. Cardiovascular disorders are associated with a depletion in ATP production, leading to the stimulation of NF-κB, which plays a critical role in the regulation of inflammatory responses. In cardiovascular diseases, NF-kB activation is associated with the production of pro-inflammatory cytokines, such as interleukin-6 (IL-6) and tumor necrosis factor (TNF), and the promotion of vascular inflammation, endothelial dysfunction, and atherosclerosis. IL-6 is a cytokine that plays a central role in the regulation of the immune response and inflammation [58]. In cardiovascular diseases, IL-6 and TNF are involved in the pathogenesis of atherosclerosis, myocardial infarction, and heart failure. Elevated levels of IL-6 or TNF are associated with an increased risk of cardiovascular events and a worse prognosis in patients with heart failure [59]. Furthermore, in cardiovascular diseases, collagen is involved in the regulation of tissue repair and remodeling, particularly in response to injury or stress. Excessive collagen deposition can lead to the development of fibrosis, impaired cardiac function, and an increased risk of adverse cardiovascular events, including heart failure. TGF-1 is a cytokine that plays a crucial role in the regulation of tissue repair and remodeling. In cardiovascular diseases, TGF-1 is involved in the regulation of cardiac fibrosis, atherosclerosis, and vascular remodeling. Elevated levels of TGF-1 are associated with increased risk of adverse cardiovascular events, including heart failure.

**Figure 2:**
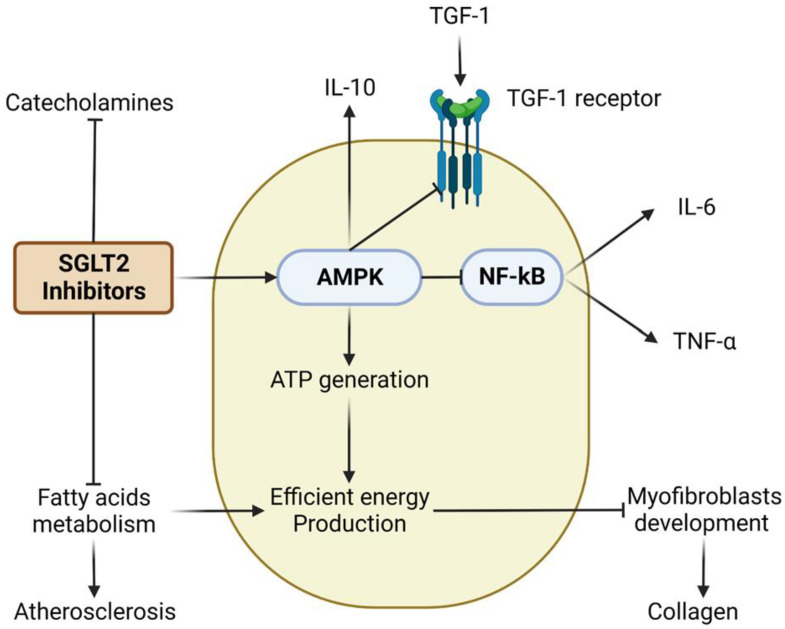
Cardiac effects of SGLT2 inhibitors.

SGLT1 and SGLT2 have received the greatest attention. The heart expresses the former (abundantly among heat failure patients). SGLT2, on the other hand, is not present in cardiac cells and functions largely in the kidney’s proximal convoluted tubule (PCT) [60]. The lack of SGLT2-R in the heart, along with the fact that antidiabetic medicines take years to decrease glucose levels and hence exhibit cardiovascular effects, makes interpreting the mechanism of action of SGLT2 inhibitors more difficult [61]. There have been a few metabolic hypotheses proposed to explain such cardiovascular advantages, including:

(1) Cardiac AMPK phosphorylation, which enhances fatty acid oxidation and hence more ATP generation [62]. SGLT2 inhibitors activate AMPK via the inhibition of Complex I of the mitochondrial respiratory chain (Figure 2). AMPK is a highly conserved enzyme that detects changes in cellular energy levels and becomes active when there is an increase in AMP/ATP or ADP/ATP ratios. The main function of AMPK is to maintain energy balance by reducing ATP-consuming processes, including the transcription of genes involved in the synthesis of fat and ribosomal proteins, and the production of cholesterol and fatty acids [63]. Meanwhile, AMPK increases metabolic pathways such as glucose and fatty acid transport, fatty acid oxidation, autophagy, mitochondrial synthesis, and oxidative metabolism to preserve ATP during times of energy deficiency [64,65,66,67,68,69].

(2) A change in heart metabolism to favor ketone bodies (KB) over fatty acids or glucose, resulting in more oxygen-efficient energy production [70].

Activation of AMPK encourages efficient energy production, leading to the regulation of collagen formation. AMPK-induced inhibition of NF-kB leads to anti-inflammatory effects via suppression of IL6 and TNF-α release and stimulation of IL-10 formation. Additionally, SGLT2 inhibitors showed an antifibrotic effect by inhibition of TGF-1 receptors.

Interestingly, SGLT2 has been shown to have a role in the inflammatory response, which may help to reduce unfavorable cardiac remodeling and enhance cardiovascular outcomes. For example, following ischemia-reperfusion damage, SGLT1 knockdown animals displayed decreased oxidative stress, resulting in less myocardial necrosis and smaller infarct size. NADPH oxidase 2 (NOX2) was downregulated, which mediated these effects [71]. Therefore, SGLT2 inhibitors have been linked to anti-inflammatory properties [72].

### 4.1. Dapagliflozin

Dapagliflozin lowered collagen production after myocardial infarction (MI) in mice by activating anti-inflammatory macrophages and suppressing myofibroblast development (Table 2). The dapagliflozin group also had a higher level of anti-inflammatory cytokine subtype 10 (IL-10) in this research [73].

### 4.2. Empagliflozin

In a dose-dependent manner, empagliflozin inhibited human fibroblast activation via transforming growth factor 1 (TGF1) (Figure 2). The empagliflozin group also had a lower level of pro-fibrotic markers (such as collagen type I 1 chain and matrix metallopeptidase 2) than the placebo group [74]. Accordingly, it was discovered that empagliflozin reduced sympathetic overdrive (i.e., catecholamine levels), a factor in neurohormonal activation and a key indicator of adverse cardiac remodeling [75]. Empagliflozin administration for 10 weeks was associated with a reduction in the formation of atherosclerotic lesions in the aorta in a high-fat diet-fed apolipoprotein E (APOE) mutant mice model. Additionally, the authors of this study noted that, while SGLT1 was expressed in all aortic samples, SGLT2 was only found in a small number of them [76].

Empagliflozin has also been shown to reduce inflammation in the kidneys. Empagliflozin substantially decreased renal production of pro-inflammatory cytokines and chemokines (including tumor necrosis factor (TNF)), urine indicators of kidney inflammation (such as IL-6), and apoptosis in a diabetes-induced rat model [77]. In this study, empagliflozin was also associated with decreased expression of pro-fibrotic genes such as TGF, collagen type IV, and fibronectin (Table 2) [78].

**Table 2:** Actions of SGLT2 inhibitors on different body systems.

	Dapagliflozin	Empagliflozin	Canagliflozin	Ertugliflozin
Cardiovascular effects	▪ Decreased collagen formation [73]. ▪ Activated IL-10 [73].	▪ Inhibited fibroblast activation [74]. ▪ Reduced sympathetic overdrive. ▪ Reduced atherosclerosis. ▪ Decreased renal production of inflammatory cytokines.	▪ Decreased TNFR1 and pro-inflammatory cytokines. ▪ Inhibited intracellular glucose metabolism. ▪ Promoted autophagy.	
Metabolic effects	▪ Increased fat burning. ▪ Aided in weight loss. ▪ Altered histone H3K9. ▪ Raised circulation levels. ▪ Elevated zinc 2 glycoprotein expression [79]. ▪ Improves diabetes-induced heart failure [77].	▪ Influenced energy expenditure. ▪ Influenced the expression of uncoupling protein 1 [80]. ▪ Regulated adipocyte metabolism [81].		
Cancer	▪ Lowered the incidence of respiratory system malignancies [82]. ▪ Inhibitory effect on colon cancer cells [83]. ▪ Suppressed tumor development by limiting glucose entrance into cancer cells [84]. ▪ Caused apoptosis in renal cancer cells [85].	▪ No connection to the occurrence of bladder or kidney cancer [86]. ▪ Anti-inflammatory and antioxidant effects lowered cancer risk [87].	▪ Preventive effect against gastrointestinal cancer. ▪ Inhibited lung cancer cell growth [88]. ▪ Reduced breast cancer incidence. ▪ Reduced breast cancer cell proliferation [88].	▪ Did not increase the risk of malignant tumors [89].
Bone metabolism	▪ No changes in bone resorption or formation [90].	▪ No changes in bone resorption or formation [91].	▪ Increased bone resorption markers [92,93]. ▪ Affected the cortical and trabecular bone microarchitecture [94]. ▪ Reduced total hip BMD [95]. ▪ Lowered estradiol levels and BMD [93].	▪ Showed no adverse effects [96].
Neuroprotective effect	▪ Lowered the clinical and electroencephalographic signs of brain seizure activity [97]. ▪ Inhibited ROS-induced neuronal death. ▪ Reduced neuroinflammation. ▪ Inhibited AChE enzyme [98].	▪ Decreased amyloid levels [99]. ▪ Reduced inflammatory mediators.		

### 4.3. Canagliflozin

In numerous inflammatory models, canagliflozin was found to have anti-inflammatory properties. Through a decrease in tumor necrosis factor receptor 1 (TNFR1) and IL-6, canagliflozin aids in the reversal of molecular processes associated with inflammation, extracellular matrix turnover, and fibrosis [100]. The reduction in TNFR1, IL-6, matrix metalloproteinase 7 (MMP7), and FN1 implies that canagliflozin aids in the reversal of inflammation, extracellular matrix turnover, and fibrosis molecular processes. Canagliflozin inhibits intracellular glucose metabolism and promotes autophagy in immune cells, resulting in anti-inflammatory actions [101].

## 5. Cardiorenal Effects of SGLT2 Inhibitors

SGLT2 inhibitors showed a greater rate of osmotic diuresis and natriuresis. Increased natriuresis and osmotic diuresis are associated with decreased renin production from the juxtaglomerular apparatus, vasoconstriction of the afferent and vasodilation of the efferent arteriole at the glomeruli level, and subsequently decreased filtration rate and intraglomerular hydrostatic pressure (Table 2) [102]. SGLT2 inhibitor-treated patients exhibited a −3.8/−1.5 mmHg systolic/diastolic blood pressure (BP) change from baseline to placebo-treated patients [103]. SGLT2 inhibitors lowered glycated hemoglobin by 0.46 percent when compared to a placebo, and this blood glucose reduction was connected to a lower risk of all fatal and non-fatal events except stroke [104]. The fact that increased urine volume returns to pre-treatment levels after three months and that BP reduction lasts almost four years suggests that osmotic diuresis is not the main mechanism causing BP reduction. Other proposed mechanisms include nephron remodeling that reduces arterial stiffness [105], relaxation of vascular smooth muscle cells due to a negative sodium balance [106], reduction in sympathetic drive [107], and weight loss independent of fluid contraction caused by glycosuria [103].

Overall, diuresis caused by SGLT2 inhibitors may result in a considerable yet clinically relevant decline in systolic and diastolic blood pressure of roughly 4 and 1 mmHg, respectively. This BP-lowering effect does not appear to be accompanied by an elevated risk of hypotensive episodes such as syncope in T2DM patients with hypertension who are already taking appropriate antihypertensive medication [86]. However, in hypertensive patients on conventional diuretics, extra caution should be used due to the increased risk of volume depletion or acute renal damage seen with SGLT2 [108]. Along with lowering glycated hemoglobin, fasting and postprandial plasma glucose levels, body weight, and blood pressure, SGLT2 inhibitors also lower the risk of a number of cardiovascular and renal complications without raising the risk of hypoglycemia [109].

## 6. Metabolic Effects of Antidiabetic Drugs on Adipocytes

### 6.1. The Role of Adipose Tissue in Diabetic Complications

Diabetes mellitus is a metabolic disorder that tends to be inflammatory. TNF, IL1, IL6, IL18, IL8, intracellular and vascular cell adhesion molecules, matrix metalloproteinases, and monocyte chemoattractant protein 1 (MCP-1) are all upregulated in diabetes and have been connected to its associated complications. Furthermore, pancreatic cell malfunction is linked to cellular inflammatory responses [110]. Since inflammatory reactions play a significant role in peripheral insulin resistance and diabetes, restoring the inflammatory milieu to a normal physiological state is a relatively new goal of diabetes treatment [111].

Brown and white fat, which are composed of brown and white adipocytes, respectively, are the two main forms of adipose tissue. White adipocytes are large, spherical cells with a single large lipid drop in the middle and a thin cytoplasm around a nucleus [112]. Triacylglycerols, the body’s major source of energy, are stored in white adipocytes. These cells also have an endocrine role, secreting leptin, adiponectin, and proinflammatory cytokines, among other things. Brown adipocytes are also tiny cells with a lot of mitochondria in their cytoplasm and a lot of lipid droplets [113]. The main physiological role of brown adipocytes is to control thermogenesis. These white adipocytes, often referred to as beige adipocytes, have the same qualities and functions as brown adipocytes but are found in white adipose tissue [114].

By diminishing peripheral insulin sensitivity and increasing gluconeogenesis, visceral fat accumulation has been demonstrated to play a significant role in the pathogenesis of insulin resistance [115]. Additionally, visceral fat is a metabolically active tissue that secretes adipocytokines and inflammatory indicators [116]. Subcutaneous adipose tissue is more strongly linked to insulin resistance than visceral fat. As a result, the development of insulin resistance and diabetes mellitus is influenced by both visceral and subcutaneous adipose tissue [117].

Adipokines are bioactive molecules generated by adipose tissue that have a role in the inflammatory response’s control. Since the discovery of leptin in 1994, hundreds of novel adipokines have been found, all of which are generated by adipocytes and are involved in fatty acid metabolism and glucose homeostasis [118]. These adipocyte-derived active molecules may compromise normal insulin signaling, resulting in insulin resistance and diabetes. Several adipokines have hypoglycemic and insulin-sensitivity-improving properties. Adiponectin enhances insulin sensitivity by lowering inflammation, whereas cytokines diminish it by generating inflammation. Adipocyte malfunction and decreased metabolism are closely related to insulin resistance and diabetes, even though their role in insulin sensitivity/resistance is complex and poorly understood. Adipokines control insulin sensitivity and secretion, contributing to the etiology of diabetes [119].

M2 macrophages are a type of immune cell that is involved in tissue repair and anti-inflammatory responses. In adipose tissue, M2 macrophages play a role in regulating adipocyte metabolism by promoting adiponectin secretion and reducing inflammation [120]. Histone H3K9 is a modification of histone proteins that can affect gene expression. Recent studies have shown that changes in histone H3K9 modifications in adipocytes can affect adipocyte metabolism and contribute to the development of metabolic disorders [121]. Zinc glycoprotein, a protein found in adipose tissue, has been shown to regulate insulin sensitivity and glucose metabolism. Studies have shown that zinc glycoprotein levels are decreased in obesity and type 2 diabetes, and supplementation with zinc can improve insulin sensitivity and glucose metabolism. Adiponectin and M2 macrophages play important roles in promoting fat breakdown and reducing inflammation in adipose tissue [122].

### 6.2. Adipocyte Metabolism the Presence of SGLT2 Inhibitors

#### 6.2.1. Dapagliflozin

**Figure 3:**
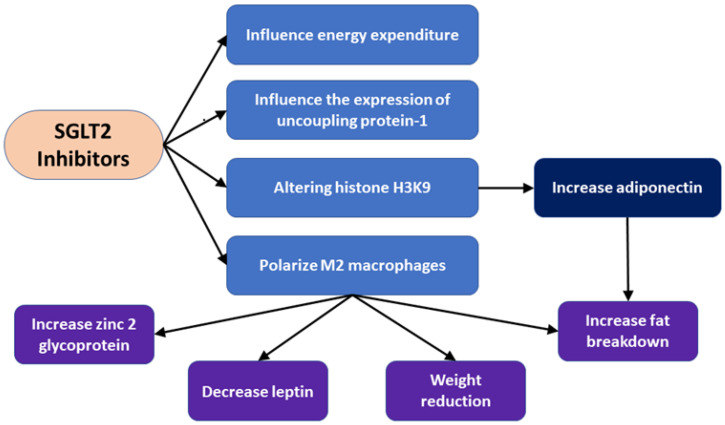
Effect of SGLT2 inhibitors on adipose tissue.

Dapagliflozin increases fat burning to aid in weight loss in T2DM patients. By directly altering histone H3K9, dapagliflozin boosts the synthesis of 3 hydroxybutyrate, which in turn upregulates adiponectin in adipocytes and enhances insulin sensitivity in obese diabetic rats. SGLT2 inhibition raises circulation levels and zinc 2 glycoprotein gene expression (encoded by the AZGP1 gene) in T2DM patients (Figure 3), improving insulin sensitivity [79] (Table 2).

Dapagliflozin improves diabetes-induced heart failure in diabetics by increasing epicardial fatty tissue and decreasing TNF levels. Furthermore, canagliflozin was discovered to enhance adipocyte function by decreasing plasma leptin levels while boosting adiponectin levels [77], demonstrating that dapagliflozin induces weight reduction without affecting HDL levels significantly. As a result, SGLT2 inhibition seems to lower blood glucose not just through glucosuric induction but also through increased adiponectin expression and insulin sensitivity [123]. Furthermore, SGLT2 inhibition decreases weight, blood pressure, and has cardiac and renal benefits in those with preserved renal function. However, SGLT2 inhibitors may be terminated due to adverse effects such as vaginal infections, polyuria, dehydration, hypotension, and risk of fractures [116].

#### 6.2.2. Empagliflozin

It has long been known that SGLT2 inhibitors have an impact on adipocyte metabolism [81]. Regarding adipocytes and adipokine expression, SGLT2 inhibition has been shown to influence energy expenditure, heat generation, and the expression of uncoupling protein 1 (Table 2). By polarizing M2 macrophages (Figure 3), it was also shown that blocking SGLT2 with empagliflozin stimulates fat burning and enhances insulin sensitivity in obese mice’s white adipose tissue [80]. The effects of SGLT2 inhibition on adipose tissue and its circuits with the brain, which are altered by SGLT2 inhibition in obese mice, are substantially responsible for the weight reduction associated with it. In animal models, SGLT2 inhibitors have had varying effects on brown and white fat tissue, as they may increase or decrease fat consumption and burning in brown fat.

SGLT2 inhibitors influenced energy expenditure, uncoupling protein 1 expression, and polarized M2 macrophages. These effects improved adiponectin levels and led to increased fat breakdown, weight reduction, and increased zinc 2 glycoprotein expression.

## 7. SGLT2 Inhibitors and Cancer

SGLT2 inhibitors could have significant clinical benefits in treating cancer. Cancer cells typically rely on glucose utilization for energy and substrates for RNA and DNA synthesis, and SGLT2 inhibitors can selectively block glucose uptake by cancer cells. SGLT2 inhibitors are normally used to treat diabetes and cause glucosuria, natriuresis, and uricosuria. They are well tolerated and safe, and have been found to be effective in treating heart failure in patients without diabetes. The expression of SGLT2 has been confirmed in various types of cancer cells, making them a promising target for therapy [124]. recent evidence suggests that SGLT2 inhibitors may have potential as an anti-cancer therapy. The anticancer activity of SGLT2 inhibitors has been demonstrated in such cancers as liver, pancreatic, prostate, bowel, lung, and breast cancer [124]. Some types of cancer cells express SGLT2, and SGLT2 inhibitors have been shown to induce apoptosis or inhibit the proliferation of cancer cells at high concentrations in vitro [125].

Cancer cells often have an altered metabolism, including an increased reliance on glucose for energy. As a result, some anticancer drugs target glucose metabolism in an attempt to disrupt the energy supply of cancer cells. Tumor cells enhance glucose uptake across the plasma membrane via induction of GLUTs, which have different affinities for glucose and transport capacities [126], inhibiting the function of these transporters can reduce the amount of glucose available to cancer cells, potentially inhibiting their growth.

Recent research has shown that SGLT2 inhibitors may also have potential anticancer effects by blocking cellular glucose uptake, and impeding tumor growth [127]. SGLT2 inhibitor has been shown to inhibit glucose uptake by SGLT2-expressing human liver cancer cells (Huh7 and HepG2) at clinically comparable doses and in a dose-dependent manner, reduce intracellular adenosine triphosphate (ATP) levels, induce cell apoptosis, and indirectly suppress tumor angiogenesis [128]. Additionally, some research has shown that SGLT2 inhibitors could decrease mTOR and BCL gene expression and upregulate pro-apoptotic gene expression (p21).

### 7.1. Dapagliflozin

Dapagliflozin appeared to lower the incidence of respiratory system malignancies when compared to the control group, however, the difference was not statistically significant. By boosting the expression of SGLT2 in lung premalignancy and early stage lung adenocarcinoma [82], dapagliflozin may be able to inhibit glucose transport and hence reduce the rate at which cancer cells proliferate. Dapagliflozin may increase the overall risk of malignant tumors, with digestive tract malignancy being more probable. However, no statistically significant difference has been observed [129].

Dapagliflozin has been demonstrated to have a possible inhibitory effect on colon cancer cells that express SGLT2 but not UDP glucuronosyltransferase family 1 member A9 (UGT1A9) [83]. Furthermore, non-metabolized dapagliflozin has been shown to suppress tumor development by limiting glucose entrance into cancer cells and exerting lethal effects [84]. As a result, it is unclear if the dapagliflozin-induced rise in digestive tract malignancy is significant.

SGLT2 is highly expressed, participating in glucose absorption in prostate cancer, according to prior research [130]. Hence, SGLT2 inhibitors may decrease tumor development by lowering glucose uptake and altering glycolysis. Dapagliflozin has been shown to cause apoptosis in renal cancer cells [85].

### 7.2. Empagliflozin

It was suggested that empagliflozin was linked to a higher incidence of bladder cancer. Nonetheless, the validity of the data used in this study was questioned, and the rectified data revealed that empagliflozin may not be connected to bladder cancer [131]. Although not statistically significant, empagliflozin may reduce the overall risk of tumors as well as the incidence of malignant tumors in the urinary system. A review of data from 20 placebo- and empagliflozin-controlled trials revealed no indication of a connection between the medication and the occurrence of bladder or kidney cancer, according to an observational, prospective follow-up study [86].

Anti-inflammatory and antioxidant effects have also been linked to a lower risk of cancer. Furthermore, it was found that empagliflozin dramatically raised the incidence of malignant tumors, particularly those of the digestive system, when compared to a placebo [87]. However, an analysis that includes 15 randomized phase I–III trials, four extension studies, and placebo-controlled investigations has identified no association between the safety of empagliflozin and T2DM patients with a malignant tumor. It is still up for debate whether or not empagliflozin increases the risk of cancer [132] (Table 2).

### 7.3. Canagliflozin

**Figure 4:**
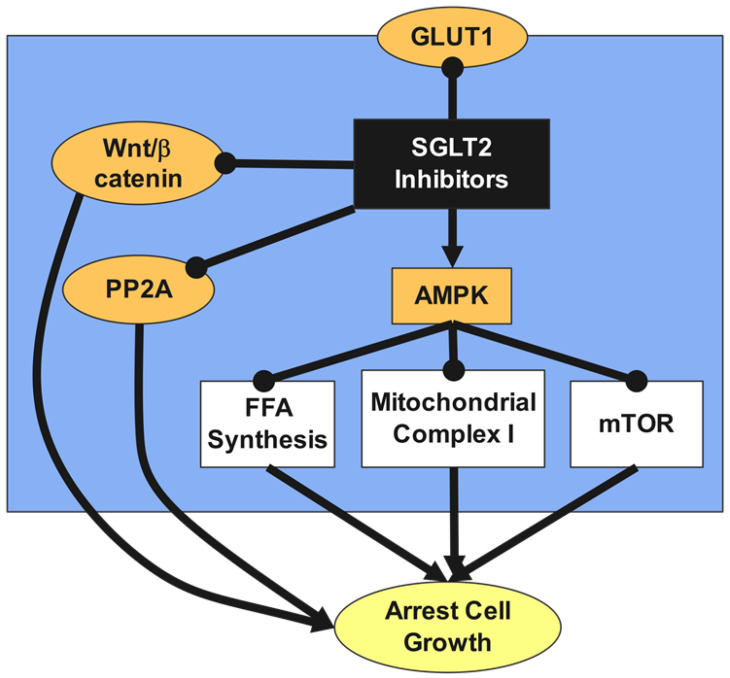
Anticancer effects of SGLT2 inhibitors.

Canagliflozin inhibits the translocation of β-catenin from the cytoplasm to the cell nucleus and enhances its proteasomal degradation, leading to a reduction in HCC growth [133]. Canagliflozin inhibits not only SGLT2 but also other glucose transporters such as GLUT1, which is overexpressed in HCC cells. Canagliflozin’s anticancer effect is not solely due to inhibiting glucose influx but also involves direct inhibition of protein phosphatase 2A (PP2A) activity [124]. The Wnt/β-catenin signaling pathway plays a crucial role in HCC development, and its excessive activation is a predisposing factor for HCC (Figure 4). Canagliflozin inhibits the Wnt/β-catenin signaling pathway, leading to reduced HCC growth [134].

Canagliflozin inhibits cancer cell proliferation and metabolic reprogramming by downregulating mitochondrial complex I and α subunit of ATP synthase F1, leading to inhibition of oxidative phosphorylation and reduction of cellular ATP concentration, which increases the AMP/ATP ratio and activates adenosine monophosphate-activated protein kinase (AMPK) [135]. AMPK activation inhibits fatty acid synthesis, resulting in the suppression of lipogenesis and cancer cell proliferation. Canagliflozin was shown to strongly and dose-dependently inhibit mitochondrial complex I without affecting complex II, and its effects were found to be dose-dependent and specific [84].

Canagliflozin increases lipid peroxidation and leads to ferroptosis. This mechanism is being investigated as a potential treatment for cancer. Canagliflozin-induced AMPK activation leads to cell cycle arrest in the G2/M phase of liver cancer cells (Figure 4). Canagliflozin strongly inhibits oxidative phosphorylation and reduces ATP production, resulting in the inhibition of mTOR, which suppresses cell proliferation, inhibits the cell cycle in the G1 phase, and induces apoptosis in cancer cells. These mechanisms have been confirmed in breast and pancreatic cancer cells [136,137].

Canagliflozin was also found to have a preventive effect against gastrointestinal cancer. SGLT2 inhibitors were not significantly related to any cancer type, according to a meta-analysis of 27 studies [138]. This meta-analysis found no significant link between SGLT2 inhibitors and the overall risk of cancer in T2DM patients or drug duration. Canagliflozin was discovered to inhibit lung cancer cell growth by preventing mitochondrial-complex-I-supported respiration [88]. By reducing glycolysis and angiogenic activity (Figure 4), canagliflozin may directly prevent liver cancer development (Table 2). Our research found that canagliflozin may raise the risk of breast cancer when compared to a placebo, although there was no statistically significant difference. Breast cancer incidence in canagliflozin intervention groups was similar to that in non-canagliflozin intervention groups, and both were reduced, according to studies [139]. Canagliflozin reduced breast cancer cell proliferation by increasing AMPK phosphorylation and reducing phosphorylation of 70 kDa ribosomal protein S6 kinase 1, which disrupts the cell cycle and causes death [88].

### 7.4. Ertugliflozin

Ertugliflozin did not vary statistically from other hypoglycemic drugs or placebo alone and did not significantly increase the risk of any particular tumors when compared to other SGLT2 inhibitors [89]. The aforementioned may be caused by a small test sample size, according to certain theories [139]. A pooled study of seven randomized controlled trials found that ertugliflozin did not significantly differ from placebo or other active hypoglycemic drugs in the risk of malignancies (Table 2) [140].

SGLT2 inhibitors hinder the proliferation of cancer cells and their metabolic reprogramming through a variety of means, including downregulating the Wnt/β-catenin pathway, activating AMPK, inducing ferroptosis, and suppressing mTOR. Inhibition of glucose transport into the cancer cells will reduce their proliferation. Inhibition of mitochondrial complex I and synthesis of free fatty acids and reduced expression of PP2A could also arrest tumor cellular growth.

## 8. Effect of SGLT2 Inhibitors on Bone Metabolism and Fracture Risk

Bone metabolism and turnover are essential processes for maintaining bone health and homeostasis. Bones are dynamic tissues that constantly undergo changes in response to mechanical stresses, hormones, and other factors. Bone metabolism is the process by which bones are formed, maintained, and remodeled throughout life. Bone turnover refers to the continuous process of bone resorption and formation, which is tightly regulated by multiple factors. The two main types of cells involved in bone metabolism are osteoblasts and osteoclasts [141]. Osteoblasts are responsible for bone formation, while osteoclasts are responsible for bone resorption. These cells work in a coordinated manner to maintain a balance between bone formation and resorption, which is essential for maintaining bone strength and structure. The process of bone turnover is regulated by several hormones and factors, including vitamin D3, parathyroid hormone (PTH), fibroblast growth factor (FGF), phosphate, sodium, and glucose [142].

Vitamin D3, also known as cholecalciferol, is a fat-soluble vitamin that plays a crucial role in the regulation of calcium and phosphate metabolism, which are essential for bone formation and maintenance. It stimulates the absorption of calcium and phosphate from the intestines and also promotes the resorption of calcium and phosphate from bone. Low levels of vitamin D3 can lead to impaired bone mineralization and bone loss, which can contribute to osteoporosis. PTH is a hormone secreted by the parathyroid glands that play a key role in regulating calcium and phosphate metabolism. It stimulates the resorption of calcium and phosphate from bone and also enhances the production of vitamin D3 by the kidneys, which in turn promotes calcium and phosphate absorption from the intestines. High levels of PTH can lead to bone loss, while low levels can result in impaired bone mineralization [143].

FGF is a family of proteins that play important roles in bone metabolism and turnover. FGF23, for example, is produced by osteocytes and regulates phosphate metabolism by reducing the expression of sodium-dependent phosphate transporters in the kidneys. High levels of FGF23 can lead to hypophosphatemia, which can contribute to bone loss. Phosphate and sodium are essential minerals that play important roles in bone metabolism and turnover. Phosphate is a key component of hydroxyapatite, the mineral matrix of bone, while sodium plays a role in regulating bone cell function. Imbalances in these minerals can lead to bone loss and other bone disorders [144].

Glucose levels can also affect bone metabolism and turnover. High levels of glucose can lead to the formation of advanced glycation end products (AGEs), which can accumulate in bone and impair bone quality. Diabetes has been linked to an increased risk of osteoporosis and bone fractures [145]. Patients with T2DM are more likely to suffer hip fractures, which are the most serious of all osteoporotic fractures, as well as limb fractures such as leg or ankle fractures [146]. In individuals with T2DM, canagliflozin and dapagliflozin have been linked to an increased risk of bone fracture (Table 2). T2DM has been associated with anomalies in the trabecular and cortical bone microarchitecture of the femur and axial skeleton as shown in various animal studies [147]. Unfavorable cortical bone microarchitecture (increased cortical porosity) at the distal radius was seen in postmenopausal women with T2DM, with potential negative effects on bone strength. In older males with T2DM, bone strength in the cortical-rich midshaft of the radius was reduced [148]. In individuals with T2DM, bone mineral density (BMD) may stay the same, decrease, or rise. According to several studies, people with T2DM have a greater BMD. Despite having a greater baseline BMD, diabetic white women have seen an increased bone loss in the femoral neck. Increased BMD has been linked to a higher body mass index (BMI), whereas inadequate bone turnover has been linked to insulin resistance [149].

### 8.1. Potential Mechanism of SGLT2 Inhibitors on Bone Metabolism, Turnover, Microarchitecture, and Calcium and Phosphate Hemostasis

**Figure 5:**
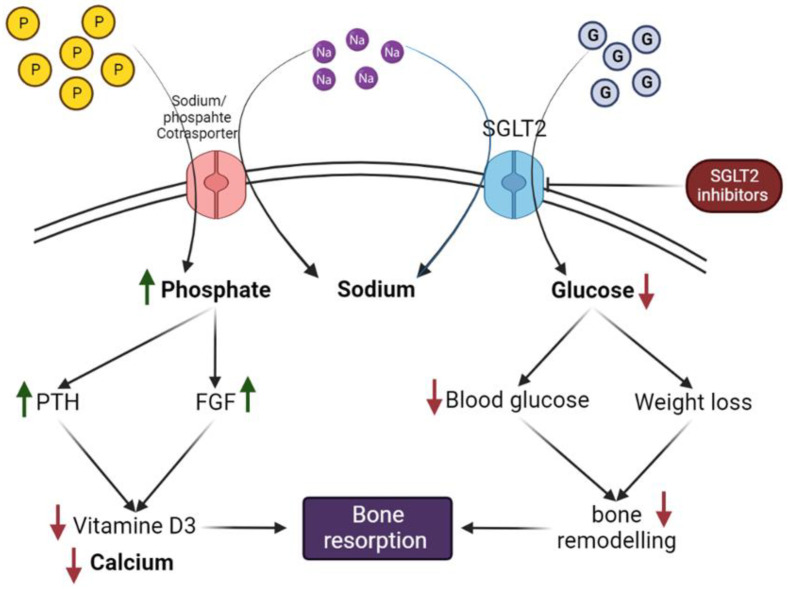
Mechanism of SGLT2 inhibitors on bone metabolism.

The impact of SGLT2 inhibitors on bone metabolism is thought to be related to a disrupted calcium and phosphate balance caused by the inhibition of the sodium–glucose cotransporter. This leads to higher levels of parathyroid hormone (PTH) and lower amounts of 1,25-dihydroxy vitamin D, which affects bone metabolism [143]. SGLT2 inhibitors may promote bone turnover indirectly through weight reduction and improve bone metabolism impairment in diabetics by lowering blood glucose levels (Figure 5). The effect of SGLT2 inhibitors on bone turnover varies depending on the medication.

The sodium–phosphate cotransporter in the proximal tubules of the kidney can carry more sodium and phosphate when SGLT2 inhibitors are present because they inhibit the sodium and glucose cotransporters. Increased phosphate absorption and urine calcium excretion will occur as a result of the salt loss. Increased levels of blood phosphate may encourage FGF, which controls systemic phosphate homeostasis and vitamin D synthesis. Increased serum FGF, therefore, induces phosphaturia and prevents the synthesis of 1,25-dihydroxyvitamin D, which is essential for preserving phosphate balance. When 1,25-dihydroxyvitamin D levels fall, there is a reduction in calcium absorption from the gastrointestinal tract, which may have an impact on bone mineralization. Through weight reduction, SGLT2 inhibitors may indirectly promote bone turnover. Diabetes-related impairment of bone metabolism may be improved by lowering blood glucose levels.

#### 8.1.1. Dapagliflozin

After 50 and 102 weeks of dapagliflozin therapy, no changes in bone resorption or formation were observed. Dapagliflozin showed no discernible changes in BMD in the lumbar spine, femoral neck, or total hip [90]. Weight loss could only explain a portion of the decrease in BMD; therefore, more research is needed to determine if SGLT2 inhibitors impact BMD alterations following treatment with other medicines. Dapagliflozin slightly elevated phosphate levels over the baseline.

#### 8.1.2. Empagliflozin

Studies have shown that empagliflozin treatment can lead to a decrease in serum phosphate levels. This may be due to the increased renal excretion of phosphate or to a direct effect of empagliflozin on phosphate transport in the intestine [143]. A decrease in serum phosphate levels could potentially have a positive effect on bone metabolism, as high levels of serum phosphate have been associated with increased bone resorption and decreased bone mineral density. Empagliflozin may also affect bone turnover and microarchitecture through its effect on glucose metabolism. Diabetes is associated with an increased risk of bone fractures, which may be due in part to the negative effects of hyperglycemia on bone turnover and microarchitecture. Empagliflozin treatment has been shown to improve glucose metabolism and reduce markers of inflammation, which could potentially improve bone health [144]. One potential mechanism by which empagliflozin may affect bone metabolism is through the regulation of the receptor activator of nuclear factor kappa-B ligand (RANKL) and osteoprotegerin (OPG), which are key regulators of bone resorption. Studies have shown that empagliflozin treatment can decrease the expression of RANKL and increase the expression of OPG in bone cells, leading to a decrease in osteoclast activity and bone resorption [145].

Empagliflozin may also affect bone metabolism through its effects on the Wnt signaling pathway. Wnt signaling plays a crucial role in regulating bone formation and turnover, and empagliflozin has been shown to increase the expression of genes involved in Wnt signaling in osteoblasts. Additionally, empagliflozin has been shown to increase the expression of bone morphogenetic protein 2 (BMP-2), which is a potent inducer of bone formation and a regulator of Wnt signaling [146]. Another potential mechanism by which empagliflozin may affect bone metabolism is through its effects on fibroblast growth factor 23 (FGF23), which is a key regulator of phosphate homeostasis. Studies have shown that empagliflozin treatment can decrease FGF23 levels in diabetic patients, which may improve bone health by decreasing phosphate levels and reducing the risk of vascular calcification [147].

#### 8.1.3. Canagliflozin

Canagliflozin’s label has been updated by the US Food and Drug Administration to incorporate information concerning fracture risk and decreased bone mineral density (BMD) [148]. In male diabetic DBA/2J mice, canagliflozin significantly increased the blood levels of cross-linked C-terminal telopeptides of type I collagen, a marker of bone resorption [92]. Another animal study found that canagliflozin dramatically increased bone resorption [93]. Type I collagen may underestimate bone resorption in diabetic individuals because diabetes is linked to a decrease in enzymatic cross-links, which are important for the stability and strength of collagen fibers in bone. As a result, it is still uncertain if various SGLT2 inhibitors cause higher bone resorption in the clinic [149]. Canagliflozin may negatively affect bone microarchitecture, which may be attributed to diabetes-related declines in bone structural toughness and strength. Canagliflozin treatment for 10 weeks significantly affected the cortical and trabecular bone microarchitecture in male diabetic DBA/2J mice, resulting in reduced bone density in the femur and vertebrae [94]. In the femur of non-diabetic mice, canagliflozin decreased trabecular bone volume percentage, trabecular number, and trabecular tissue mineral density while increasing trabecular spacing [150]. Another animal study indicated that glycemic control was a significant contributor to the deterioration of bone toughness and the structural strength in the femur and vertebral bodies [151]. Furthermore, neither the osteoblast nor the osteoclast cell lines exhibited any evidence of SGLT2 expression [152] (Table 2).

A reduction in total hip BMD brought on by canagliflozin may be partially attributed to weight loss. Results from a placebo-controlled, phase III clinical investigation including people with T2DM aged 55 to 80 years showed that treatment with canagliflozin for 104 weeks was associated with a reduction in BMD in the whole hip, but not in the femoral neck, lumbar spine, or distal forearm [95]. Furthermore, lowered estradiol levels may have a role in BMD loss, as canagliflozin medication has been demonstrated to lower estradiol levels in female patients, albeit the mechanism is unknown [93]. Urinary calcium loss and fibroblast growth factor 23 (FGF23) levels were observed to be greater in diabetic mice treated with canagliflozin.

To reabsorb glucose and phosphate, sodium-dependent cotransporters compete for the sodium electrochemical gradient. SGLT2 inhibitors may inhibit sodium–glucose cotransporter, enabling sodium–phosphate cotransporter to carry more phosphate in the renal proximal tubules [153].

The loss of sodium would lead to an increase in phosphate reabsorption and calcium excretion in the urine. Additionally, elevated blood phosphate levels may promote FGF23, which regulates phosphate homeostasis and vitamin D synthesis. In turn, this leads to phosphaturia and prevents the synthesis of 1,25-dihydroxyvitamin D, which is necessary to keep the phosphate balance. Reduced 1,25-dihydroxyvitamin D levels were associated with decreased calcium absorption from the digestive tract, which probably prevented skeletal mineralization [154]. In response to a drop in calcium concentration brought on by urinary calcium excretion, secondary hyperparathyroidism occurs. Recently, it was shown that taking canagliflozin increases phosphate, FGF23, and PTH levels, while it decreases 1,25-dihydroxyvitamin D levels. This may have detrimental effects on bone health [155,156,157,158,159]. The rise in phosphate, FGF23, and PTH levels, as well as the reduction in 1,25-dihydroxyvitamin D levels, were all minor and occurred within the first few hours/days after treatment. It is unclear if these alterations will last following long-term SGLT2 inhibitor treatment.

## 9. Cognitive Impairment and SGLT2 Inhibitors

Diabetes mellitus type 2 is linked to a higher risk of cognitive impairment, which can impact many cognitive areas. The processes behind the development of cognitive impairment in diabetic individuals are yet unknown [160]. Although more study into potential candidate processes is needed, present evidence shows that the etiology of cognitive impairment in diabetic individuals may be a mix of vascular and neurodegenerative damage [161]. This process is believed to be influenced by deficiencies in intracellular signaling, mitochondrial metabolism, oxidative stress, and insulin receptor sensitivity. Given the presence of SGLT2 in the central nervous system, current evidence suggests that SGLT2 inhibitors, in addition to having other beneficial metabolic effects and reducing cardiovascular risk in heart failure patients regardless of diabetes mellitus status, may also have neuroprotective properties [72].

### 9.1. SGLTs in Brain

SGLTs are widely expressed in the brain, especially in the cerebellum, hippocampus, frontal cortex, caudate nucleus, putamen, amygdala, parietal cortex, and paraventricular nucleus of the hypothalamus. However, they appear to be poorly expressed in the brain stem [162]. The *SLC5A* gene family encodes for sodium-dependent glucose transporters (SGLTs) and other solute carriers. From the family of *SLC5A* in the human brain, several members have been identified: SMIT1 (*SLC5A3*), SGLT3 (*SLC5A4*), SGLT4 (*SLC5A9*)**,** SGLT6, now known as SMIT2 (*SLC5A11*), SMVT (*SLC5A6*), CHT1 (*SLC5A7*), SMCT1 (*SLC5A8)*, and SMCT2 (*SLC5A12*)**.**

### 9.2. Neuroprotective and Neuro-Cognitive Effects of SGLT2 Inhibitors

Recent studies have suggested that SGLT2 inhibitors may have neuroprotective effects on chronic brain diseases such as Parkinson’s disease (PD) [163]. It was discovered that SGLT2 inhibitors reduce glucose intake in neurons, lowering membrane excitability and depolarization in a mouse model of epilepsy [164]. Additionally, it was found that SGLT2 inhibitors have a neuroprotective impact by reducing antioxidants, anti-inflammatories, and antiapoptotic processes, which reduce stroke risk variables such as glucose levels, insulin resistance, triglycerides, and fat mass in the body [165]. Post-mortem human studies on injured brain tissue found a large increase in SGLT1 and SGLT2 [165].

SGLT2 inhibitors have been shown to improve cognitive performance in obese and type 2 diabetic rats in recent studies. SGLT2 inhibitors showed a potential reduction in brain oxidative stress and oxidative damage in db/db mice, which is followed by a decrease in both cerebral NADPH oxidase and cerebral superoxide [166]. Additionally, SGLT2 inhibitors increase the production of brain-derived neurotrophic factor (BDNF), a regulator of synaptogenesis and synaptic plasticity that is often reduced in T2DM but is believed to be essential for learning and memory [167]. Additionally, it has been demonstrated that SGLT2 inhibitors enhance insulin sensitivity in the brains of obese rats by reducing brain inflammation, brain apoptosis, and brain oxidative stress. This has the added benefit of enhancing mitochondrial brain function and significantly enhancing hippocampus synaptic plasticity [168].

#### 9.2.1. Dapagliflozin

Dapagliflozin, in particular, lowers the clinical and electroencephalographic signs of brain seizure activity considerably [97]. Dapagliflozin significantly reduced neuronal oxidative stress by reducing lipid peroxide, allowing the DJ-1/Nrf2 pathway to be restored [169]. Furthermore, dapagliflozin inhibited ROS-induced neuronal death and increased the expression of glial cell-derived neurotrophic factor (GDNF) and the phosphoinositide 3-kinase (PI3K)/ (protein kinase B) AKT/Glycogen synthase kinase (GSK-3) (Ser9) pathway [170]. It also reduced neuroinflammation by inhibiting the activation of the NF-κB pathway and lowering TNF levels [169]. Dapagliflozin revealed anti-inflammatory and antioxidant characteristics, as well as an increasing impact on brain mitochondria, in a model of obese rats produced by a high-fat diet [170]. In the obese insulin resistance group, the combination of dapagliflozin and vildagliptin also reduced cognitive deterioration, probably through comparable mechanisms (Table 2). Only dapagliflozin increased hippocampus synaptic plasticity when the two medications were compared [171]. There is growing evidence that SGLT2 inhibitors may affect pathognomonic dementia mechanisms such as the loss of cholinergic neurons, the extracellular and intracellular buildup of beta-amyloid and neurofibrillary tangles in the brain, and mammalian target of rapamycin (mTOR) hyperactivation in addition to their direct effects on the CNS [172]. It was investigated that molecular interactions between acetylcholinesterase (AChE) and SGLT2 by correctly placing dapagliflozin inside the catalytic sites of both enzymes, indicating that dapagliflozin might operate as a powerful dual inhibitor of both enzymes [98].

#### 9.2.2. Empagliflozin

It was discovered that empagliflozin decreases amyloid levels in the brain and hippocampus of empagliflozin-treated APP/PS1xd/DB mice, resulting in improved cognitive performance and, in particular, memory impairment [99]. The potential benefits of empagliflozin against T2DM-induced cognitive impairment have recently been investigated in high-fructose-diet-induced hyperglycemic rats. Empagliflozin nanoparticles demonstrated a significant decline in inflammatory mediators, oxidative stress, p-tau, and amyloid-beta levels when compared to empagliflozin when the effects of the two drugs on neurological polypeptides, proinflammatory cytokines, amyloid-beta, and oxidative parameters were assessed [173]. Furthermore, mTOR hyperactivation causes a rapid loss in brain function in rats, encouraging extensive amyloid plaque deposition, increased blood–brain barrier (BBB) permeability, and tau protein hyperphosphorylation. In this case, SGLT2 inhibitors might restore mTOR to a normal level of activity, halting the onset or progression of Alzheimer’s disease [174].

## 10. Conclusions

In conclusion, SGLT2 inhibitors have been shown to have potential cardiovascular benefits, including reducing collagen production, suppressing fibroblast activation, reducing sympathetic overdrive, and reducing blood pressure. These inhibitors have also been associated with weight loss independent of fluid contraction caused by glycosuria. However, the exact molecular mechanisms by which SGLT2 inhibitors exert their cardiovascular benefits are not yet fully understood. Further research is needed to elucidate the mechanisms of action of these drugs, particularly regarding their effects on ATP, inflammatory pathways, and fibrosis. Future research should focus on investigating the long-term cardiovascular effects of SGLT2 inhibitors and whether they can be used as a therapeutic option for preventing or treating cardiovascular disease. Additionally, more studies are needed to understand the optimal dosing and duration of treatment for SGLT2 inhibitors to maximize their cardiovascular benefits.

Adipose tissue plays a critical role in the pathogenesis of insulin resistance and diabetes by secreting adipokines and inflammatory cytokines, and by accumulating visceral fat. The discovery of novel adipokines, such as adiponectin, and their potential to improve insulin sensitivity presents a promising target for future research. SGLT2 inhibitors such as dapagliflozin and empagliflozin have been shown to have a significant impact on adipocyte metabolism, energy expenditure, and insulin sensitivity, resulting in weight loss and improved glycemic control in patients with type 2 diabetes. However, these drugs may also have adverse effects that need to be monitored closely, such as the increased risk of infections, dehydration, and hypotension. Future research could explore the potential of combining SGLT2 inhibitors with other therapies to maximize their benefits while minimizing their side effects. Additionally, further investigation is needed to fully understand the mechanisms underlying SGLT2 inhibition’s effects on brown and white fat tissue and to develop personalized treatment strategies for patients with diabetes.

SGLT2 inhibitors have shown promise as a potential treatment for cancer. By blocking glucose uptake, they can selectively target cancer cells, which rely on glucose for energy and survival. Canagliflozin and dapagliflozin, two commonly used SGLT2 inhibitors, have been found to inhibit cancer cell proliferation and metabolic reprogramming through various mechanisms, including downregulating the Wnt/β-catenin pathway, inhibiting oxidative phosphorylation, activating AMPK, inducing ferroptosis, and suppressing mTOR. While some studies have shown that SGLT2 inhibitors may increase the risk of certain types of cancer, such as breast cancer, the overall evidence suggests that they are safe and well tolerated. Future research should focus on identifying the optimal dosages and treatment durations of SGLT2 inhibitors in different types of cancer, as well as investigating their potential synergistic effects with other cancer treatments. Additionally, further studies are needed to elucidate the molecular mechanisms underlying the anticancer effects of SGLT2 inhibitors, as well as to identify new biomarkers that can predict their efficacy in cancer patients.

Bone metabolism and turnover are crucial processes for maintaining bone health and homeostasis. T2DM patients are at an increased risk of developing osteoporosis and bone fractures, which can be attributed to various factors, including the disruption of calcium and phosphate balance. SGLT2 inhibitors have been shown to have an impact on bone metabolism, and their effects on bone turnover vary depending on the medication. SGLT2 inhibitors may promote bone turnover indirectly through weight reduction and improve bone metabolism impairment in diabetics by lowering blood glucose levels. However, some studies have reported an increased risk of bone fracture in T2DM patients who are using SGLT2 inhibitors. Therefore, further research is needed to understand the potential mechanisms of SGLT2 inhibitors on bone metabolism, turnover, microarchitecture, and calcium and phosphate homeostasis, as well as the long-term effects of SGLT2 inhibitors on bone health in T2DM patients. Future research should also focus on identifying patients who are at a higher risk of developing bone fractures while using SGLT2 inhibitors and developing strategies to minimize this risk.

Diabetes mellitus type 2 is linked to cognitive impairment, and the processes underlying its development are not well understood. Current evidence suggests that cognitive impairment in diabetic individuals may result from a mix of vascular and neurodegenerative damage, which can be influenced by deficiencies in intracellular signaling, mitochondrial metabolism, oxidative stress, and insulin receptor sensitivity. SGLT2 inhibitors, which are used to manage diabetes and have other beneficial metabolic effects, have been found to have potential neuroprotective properties due to the presence of SGLT2 in the central nervous system. SGLT2 inhibitors have been shown to improve cognitive performance in both animal models and humans with diabetes. Dapagliflozin, in particular, has been found to have anti-inflammatory and antioxidant properties and has been shown to reduce clinical and electroencephalographic signs of brain seizure activity. Future research may explore the role of SGLT2 inhibitors in reducing the risk of cognitive impairment and improving cognitive function in individuals with diabetes, particularly in those at high risk of developing dementia.

## Figures and Tables

**Table 1:** Mechanisms of antidiabetic drugs.

Antidiabetic Drug	Mechanism of Action
α-Glucosidase inhibitors	▪ Affected the rate of digestion of complex carbohydrates and disaccharides. ▪ Competitive and irreversible inhibition of glucosidases.
Biguanides	▪ Little influence on insulin release. ▪ Blocked complex I of the electron transport chain. ▪ Stimulation of AMP-activated protein kinase (AMPK) sensitive signaling.
Sulfonylureas	▪ Affected sulfonylurea receptor 1 (SUR 1). ▪ Closure of ATP-dependent potassium channels.
Meglitinides	▪ Insulin secretagogues. ▪ Closure of ATP-dependent potassium channels.
GLP-1 Agonists	▪ Activated adenylate cyclase and cAMP production in pancreatic cells.
PPAR-γ agonists	▪ Controlled adipocyte cell development. ▪ Regulated lipid and glucose metabolic pathways.
DDP4 inhibitors	▪ Boosting pancreatic insulin release.
SGLT2 inhibitors	▪ Decrease SGLT2-mediated glucose reabsorption from the proximal renal tubule.

## Data Availability

Not applicable.

## List of Abbreviations

AChE: Acetylcholinesterase; Akt: Protein kinase B; AMPK: AMP-activated protein kinase; APOE: Apolipoprotein E; ATP: Adenosine triphosphate; BBB: Blood–brain barrier; BDNF: Brain-Derived Neurotrophic Factor; BMD: Bone mineral density; BMI: Body mass index; BP: Blood pressure; CHT: Choline transporter; DDP4: Dipeptidyl peptidase 4; DM: Diabetes mellitus; FGF: Fibroblast growth factor; GDNF: Glial Cell-Derived Neurotrophic Factor; GLP-1: Glucagon like peptide 1; GSK: Glycogen synthase kinase; IL: Interleukin; KB: Ketone bodies; MCP-1: Monocyte chemoattractant protein 1; MI: Myocardial infarction; MMP: Matrix metalloproteinase; mTOR: Mammalian target of rapamycin; NIS: sodium-iodide symporter; NOX2: NADPH oxidase 2; PCT: Proximal convoluted tubule; PI3K: Phosphoinositide 3-kinase; PPAR-γ: Peroxisome proliferator-activated receptor γ; PTH: Parathyroid hormone; SGLT: Sodium glucose transporter; SGLT-R: Sodium glucose transporter receptors; SMCT: sodium-coupled monocarboxylate transporter; SMIT: sodium/myo-inositol cotransporter; SMVT: sodium-dependent multivitamin transporter; SUR 1: Sulfonylurea receptor 1; T2DM: Type 2 diabetes mellitus; TGF1: Transforming growth factor 1; TNF: Tumor necrosis factor; UGT1A9: UDP glucuronosyltransferase family 1 member A9.
